# Sex-specific normal values and determinants of infrarenal abdominal aortic diameter among non-aneurysmal elderly population

**DOI:** 10.1038/s41598-021-97209-3

**Published:** 2021-09-07

**Authors:** Fang Zhu, Banafsheh Arshi, M. Arfan Ikram, Robert J. De Knegt, Maryam Kavousi

**Affiliations:** 1 Department of Epidemiology, Erasmus University Medical Center, PO Box 2040, 3000 CA Rotterdam, The Netherlands; 2 Department of Gastroenterology & Hepatology, Erasmus University Medical Center, Rotterdam, The Netherlands

**Keywords:** Epidemiology, Aneurysm, Risk factors

## Abstract

To establish age- and sex-specific distribution of the infrarenal abdominal aortic diameters (IAD) among non-aneurysmal elderly population and to investigate the associations between traditional cardiovascular risk factors and IAD in men and women. We included 4032 participants (mean age 67.2 years; 60.4% women) from the population-based Rotterdam Study, free of cardiovascular disease, who underwent IAD ultrasound assessment between 2009–2014. Linear regression analysis was used to identify determinants of IAD. The medians (inter-quartile range) of absolute IAD and body surface area (BSA)-adjusted IAD were 17.0 (15.0–18.0) mm and 9.3 (8.5–10.2) mm for women and 19.0 (18.0–21.0) mm and 9.4 (8.6–10.3) mm for men, respectively. There was a non-linear relationship between age and IAD. IAD increased steeply with advancing age and up to 70 years. After around 75 years of age, the diameter values reached a plateau. Waist circumference and diastolic blood pressure were associated with larger diameters in both sexes. Body mass index [Effect estimate (95% CI): 0.04 (0.00 to 0.08)], systolic blood pressure [− 0.01(− 0.02 to 0.00)], current smoking [0.35 (0.06 to 0.65)], total cholesterol levels [− 0.21 (− 0.31 to − 0.11)], and lipid-lowering medication [− 0.43 (− 0.67 to − 0.19)] were significantly associated with IAD in women. Sex differences in IAD values diminished after taking BSA into account. The increase in diameters was attenuated after 70 years. Differences were observed in the associations of several cardiovascular risk factors with IAD among men and women.

## Introduction

Abdominal aortic aneurysm (AAA) is a life-threatening condition, characterized by the permanent dilatation of the abdominal aorta, which is usually asymptomatic but carries a high mortality rate due to rupture^1–3^. Aortic diameter is widely used to diagnose AAA once its maximum value exceeds either 30 mm or the normal vessel diameter by 50%^1,4^, and a lower threshold is suggested for women and some Asian populations^1,5^. Defining normal values of aortic diameter is essential for the accurate diagnosis of an AAA and management of progressive dilatation^3,6,7^.

Most recent guidelines recommend AAA screening for women and men, aged 65 years or older, who smoke or have a relevant family history^8,9^. Few large studies have evaluated age- and sex-specific diameters of the infrarenal abdominal aorta in the general population based on magnetic resonance imaging (MRI) or computed tomography (CT)^3,4,10^. Ultrasound is a well-established tool for detecting AAA in many countries as it is easy to use, noninvasive, cost-effective, and free of radiation exposure. However, there is limited information regarding infrarenal aortic diameter (IAD) reference values based on ultrasound in general European populations^11^, especially the distribution of aortic sizes for women at different ages has not been well described^5^.

While the risk factors for AAA have been explored, it remains unclear whether the risk factors for aneurysm and rupture are the same as those for the enlargement of IAD. Sex differences in the prevalence, development, and outcomes of AAA have been reported^2,12,13^. However, detailed sex-specific investigations on the determinants of larger IAD among the non-aneurysmal population are scarce^4,10^.

Using data from the large prospective population-based Rotterdam Study, we aimed to establish age- and sex-specific distribution of the ultrasound-assessed IAD values among non-aneurysmal older adults. We further sought to investigate the risk factors associated with IAD in women and men.

## Methods

### Study population

The current study was embedded within the Rotterdam Study (RS), a prospective population-based cohort study in the Netherlands with participants aged 45 years or over. The cohort started in 1990, and underwent three extensions in 2000, 2006 and 2016. The participants were all extensively examined at baseline and subsequent follow-up examinations that have been taking place every 3 to 6 years. The rationale and design of the study have been previously described^14^.

We conducted this study using data from the fifth visit of the original cohort (RS-I-5: 2009–2011, n = 2147), the third visit of the second cohort (RS-II-3: 2011–2012, n = 1893) and the second visit of the third cohort (RS-III-2: 2012–2014, n = 3122). 4688 participants who underwent an ultrasound examination to assess the diameter of infrarenal abdominal aorta were included. We excluded individuals with IAD ≥ 30 mm (n = 75); those with a history of cardiovascular disease (CVD) (n = 533); those with no informed consent for follow-up (n = 22), and those with incomplete information regarding CVD prevalence (n = 6). Finally, 4032 individuals were included in the current analyses. The Rotterdam Study has been approved by the Medical Ethics Committee of the Erasmus MC (registration number MEC 02.1015) and by the Dutch Ministry of Health, Welfare, and Sport (Population Screening Act WBO, license number 1071272-159521-PG). All participants provided written informed consent to participate in the study and to have their information obtained information from their treating physicians. All methods were performed in accordance with the relevant guidelines and regulations.

### Assessment of infra-renal aortic diameters

The abdominal aorta was scanned from proximal of the superior mesenteric artery in distal direction by Ultrasound (Hitachi EUB 8500). The maximum infrarenal aortic diameter above aortic bifurcation was recorded. Diameters were traced from wall to wall of the aorta in both transverse and anterior–posterior directions. We chose the diameter values in anterior–posterior direction. All measurements were performed by professional technicians under a standard protocol.

### Assessment of cardiovascular risk factors

History of cardiovascular disease was defined as the history of coronary heart disease, heart failure, and stroke and was verified from the medical records of the general practitioner. Procedures of physical examination, clinical testing, and laboratory testing were described previously in the Rotterdam study^15^. Blood pressure was measured using a random-zero sphygmomanometer on the right arm twice and the average of the two measurements was used. Hypertension was defined as a systolic blood pressure (SBP) ≥ 140 mmHg and/or a diastolic blood pressure (DBP) ≥ 90 mmHg and/or the use of blood pressure-lowering medication. Diabetes mellitus (DM) was defined as a fasting glucose level ≥ 7 mmol/L and/or the use of glucose-lowering medication. Serum total cholesterol and high-density lipoprotein (HDL) cholesterol were assessed using a standard procedure. Waist circumference (WC) was measured at the midpoint between the lower rib and the iliac crest in all participants with standing position and gentle breathing. Body mass index (BMI) was calculated as weight (kg) / (height (m))^2^. Body surface area (BSA) was calculated by using the Mosteller formula: BSA = 0.016667 × Weight (kg)^0.5^ × Height (cm)^0.5^. Information on the use of medications was obtained from home interviews and pharmacy records. Information on smoking status and alcohol consumption was obtained by home interviews. Smoking status was defined as never, former and current. Alcohol intake data were collected from the Food Frequency Questionnaire and was expressed in grams of alcohol consumption per day in this study.

### Statistical analysis

The characteristics of study population were expressed as mean ± standard deviation (SD) or numbers (percentages). We compared the mean and proportions between women and men by using a t-test and chi-square test respectively. Mean and percentiles of diameters were calculated and stratified by age (< 60, 60–64, 65–69, 70–74, 75–79, and ≥ 80 years) and sex. Kruskal–Wallis test was used to compare distributions of IAD values between the age- and sex-stratified groups.

Associations between cardiovascular risk factors and diameters were evaluated in men and women by using univariate and multivariate linear regression models. Continuous diameter values were set as an outcome. Risk factors in the model included age, WC, BMI, SBP, DBP, HDL, total cholesterol, smoking, alcohol consumption, DM, blood pressure-lowering medication, and lipid-reducing medication. The natural cubic spline function was used to specify the non-linear relation between IAD and age. The interaction between age and other covariates was checked. Model assumptions were tested and residual plots indicated that models fitted the data well.

Missing data on some covariates were present in up to 1.5% of participants. Values of waist circumference were missing for 2 participants; values of body mass index and body surface area were missing for 4 participants; values of drug use were missing for 4 participants; values of blood pressure were missing for 6 participants; values of smoking were missing for 10 participants; values of alcohol consumption were missing for 21 participants; values of cholesterol were missing for 60 participants. Five imputed datasets were generated using the Mice package in R. The Summarized estimates were calculated based on Rubin’s rule using pool function in Mice^16^. Two-sided *p* value was considered significant at *p* < 0.05. Statistical analyses were performed with the use of R version 3.6.1 (https://www.r-project.org).

### Ethical approval

The Rotterdam Study has been approved by the Medical Ethics Committee of the Erasmus MC (registration number MEC 02.1015) and by the Dutch Ministry of Health, Welfare and Sport (Population Screening Act WBO, license number 1071272–159,521-PG).

## Results

Characteristics of the study population are presented in Table 1. Among 4032 included participants, mean (SD) age was 67.2 (8.3) years and 60.4% were women. 66.1% of the population had hypertension. Men had higher WC, BSA, SBP, DBP. Women had higher HDL and total cholesterol levels, lower alcohol intake, and were less frequently smokers.

**Table 1:** Characteristics of study population.

Variables	Total (n = 4032)	Women (n = 2437)	Men (n = 1595)	*p* value
Age (years)	67.2 ± 8.3	67.4 ± 8.4	66.9 ± 8.2	0.043
Waist circumference (cm)	91.8 ± 11.6	88.1 ± 11.1	97.4 ± 10.0	< 0.001
BSA(m^2^)	1.9 ± 0.2	1.8 ± 0.2	2.0 ± 0.2	< 0.001
BMI (kg/m^2^)	27.0 ± 4.0	27.0 ± 4.4	27.0 ± 3.3	0.796
SBP (mmHg)	142.3 ± 21.9	141.4 ± 22.5	143.6 ± 20.8	0.002
DBP (mmHg)	83.8 ± 11.1	83.0 ± 11.2	85.1 ± 10.8	< 0.001
HDL cholesterol (mmol/L)	1.5 ± 0.4	1.7 ± 0.4	1.3 ± 0.4	< 0.001
Total cholesterol (mmol/L)	5.6 ± 1.0	5.8 ± 1.0	5.3 ± 1.0	< 0.001
Hypertension, n (%)	2663 (66.1)	1568 (64.4)	1095 (68.7)	0.006
Diabetes mellitus, n (%)	450 (11.2)	239 (9.8)	211 (13.2)	0.001
Blood pressure lowering medication, n (%)	1456 (36.1)	890 (36.6)	566 (35.5)	0.522
Lipid-reducing medication, n (%)	883 (21.9)	519 (21.3)	364 (22.8)	0.266
Alcohol intake (g/per day)	7.37 ± 7.9	5.65 ± 6.2	10.01 ± 9.4	< 0.001
**Smoking status, n (%)**				< 0.001
Never	1416 (35.2)	1034 (42.6)	382 (24.0)	
Former	2018 (50.2)	1096 (45.1)	922 (57.9)	
Current	588 (14.6)	300 (12.3)	288 (18.1)	

Values are presented as mean ± SD for continuous variables and numbers (percentages) for categorical variables. P-value refers to differences in baseline characteristics between women and men.

*BSA* body surface area, *BMI* body mass index, *SBP* systolic blood pressure, *DBP* diastolic blood pressure, *HDL* high-density lipoprotein.

Both absolute diameter and BSA-adjusted diameter values had a right-skewed distribution. The age- and sex-specific means and percentiles of absolute and BSA-adjusted IAD were calculated (Table 2). The medians (Interquartile range) of absolute and BSA-adjusted IAD were 17.0 (15.0–18.0) mm and 9.3 (8.5–10.2) mm, respectively, for women; and 19.0 (18.0–21.0) mm and 9.4 (8.6–10.3) mm for men. IAD distribution was different between women and men (*p* < 0.001). The overall BSA-adjusted IAD distribution for women and men was significantly different (*p* = 0.013). However, in age-stratified groups, there was only one age interval (65–69 years, *p* = 0.004) showing significant sex differences. Supplementary Figure S1 shows mean and percentiles of absolute and BSA-adjusted IAD across various age groups. With increasing age, the expansion of absolute diameter in the upper quartiles, compared to mean value and the lower quartiles, was more remarkable. Changes in mean and percentiles in BSA-adjusted diameter were relatively similar.

**Table 2:** Means and percentiles of absolute and body surface area-adjusted infrarenal aortic diameters (mm) by age among women and men.

	Age	Women						Men					
	(years)	n	mean	25th	50th	75th	95th	n	mean	25th	50th	75th	95th
Absolute IAD	All	2437	17.0	15.0	17.0	18.0	22.0	1595	19.5	18.0	19.0	21.0	24.0
	< 60	527	16.1	15.0	16.0	17.0	19.0	375	18.6	17.0	18.0	20.0	22.0
	60–64	428	16.6	15.0	16.0	18.0	20.0	285	18.7	17.0	18.0	20.0	22.0
	65–69	609	17.2	16.0	17.0	19.0	22.0	395	19.8	18.0	20.0	21.0	25.0
	70–74	428	17.8	16.0	18.0	19.0	23.0	271	20.3	18.0	20.0	22.0	26.0
	75–79	251	17.7	16.0	17.0	19.0	22.5	166	20.1	18.0	20.0	22.0	25.8
	≥ 80	194	17.4	15.0	17.0	19.0	23.0	103	20.2	18.0	20.0	22.0	24.9
BSA-adjusted IAD	All	2436	9.5	8.5	9.3	10.2	12.2	1594	9.6	8.6	9.4	10.3	12.2
	< 60	527	8.8	8.1	8.8	9.4	10.5	375	8.9	8.1	8.8	9.5	10.8
	60–64	428	9.1	8.3	9.0	9.7	11.2	285	9.0	8.4	8.9	9.5	10.7
	65–69	609	9.5	8.5	9.3	10.3	12.1	395	9.8	8.8	9.7	10.7	12.2
	70–74	427	10.0	9.0	9.8	10.7	12.8	271	10.1	9.2	10.0	11.0	12.7
	75–79	251	10.0	8.8	9.8	11.0	12.8	166	10.2	9.2	10.2	11.3	12.7
	≥ 80	194	10.3	9.1	9.8	11.3	13.1	102	10.4	9.5	10.1	11.1	13.4

*IAD* infrarenal aortic diameter, *BSA* body surface area.

**Figure 1:**
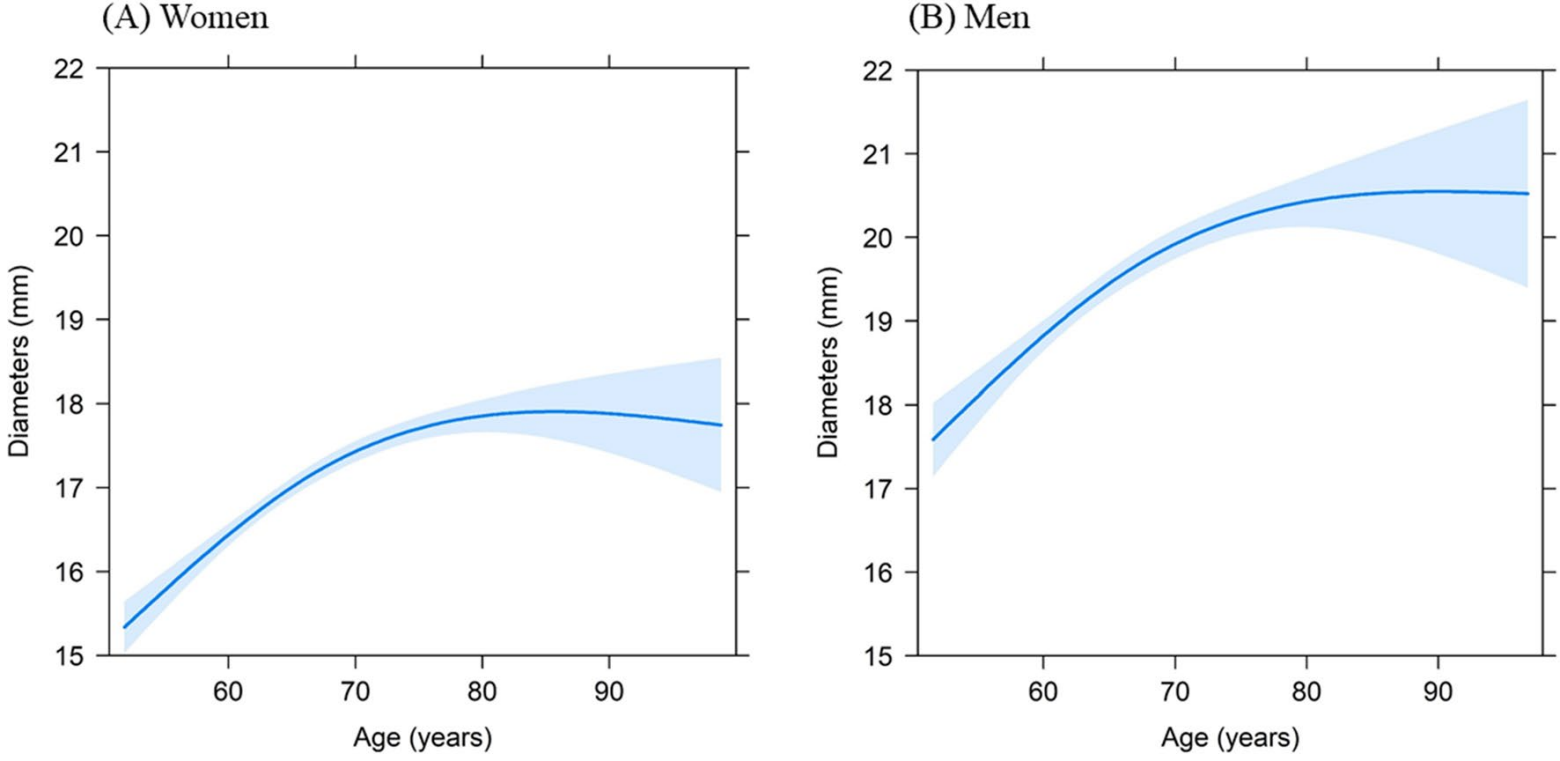
Effect plot for the association between infrarenal aortic diameters and age among women and men. Blue shaded areas represent 95% confident intervals.

The results for univariate analyses for the association between each cardiovascular risk factor with IAD are resented in Supplementary Table S1. Table 3 represents regression coefficients and 95% confidence intervals (CIs) for multivariate-adjusted linear regression models for women and men. There was a non-linear relationship between age and diameter (*p* < 0.001). The increase in diameters was attenuated after about 70 years for both women and men according to the effect plot (Fig. 1). WC [Effect estimate (95% CI): 0.02 (0.01 to 0.04) in women and 0.03 (0.01 to 0.06) in men] and DBP [0.03 (0.02 to 0.05) in women and 0.02 (0.01 to 0.04) in men] were significantly associated with absolute diameters in both sexes. BMI [0.04 (0.00 to 0.08)], SBP [− 0.01(− 0.02 to 0.00)], current smoking [0.35 (0.06 to 0.65)], total cholesterol [− 0.21 (− 0.31 to − 0.11)], and use of lipid-reducing medication [− 0.43 (− 0.67 to − 0.19)] were significantly associated with IAD in women. Diabetes and alcohol consumption were not significantly associated with IAD. Results regarding determinants of BSA-adjusted diameters are shown in Supplementary Table S2. HDL and blood pressure lowering medication showed significant associations with BSA-adjusted diameters in both sexes.

**Table 3:** Associations between cardiovascular risk factors and absolute infrarenal aortic diameter among women and men.

Risk factors	Women		Men	
	Effect estimate (95% CI)	*p* value	Effect estimate (95% CI)	*p* value
(Intercept)	11.83 (10.61 to 13.05)	** < 0.001**	13.93 (12.12 to 15.74)	** < 0.001**
Age [1st]*	4.38 (3.69 to 5.07)	** < 0.001**	5.07 (4.08 to 6.05)	** < 0.001**
Age [2nd]*	1.25 (0.43 to 2.07)	**0.003**	1.94 (0.79 to 3.10)	**0.001**
BMI	0.04 (0.00 to 0.08)	**0.048**	0.01 (− 0.06 to 0.09)	0.727
WC	0.02 (0.01 to 0.04)	**0.006**	0.03 (0.01 to 0.06)	**0.011**
DBP	0.03 (0.02 to 0.05)	** < 0.001**	0.02 (0.01 to 0.04)	**0.011**
SBP	− 0.01 (− 0.02 to 0.00)	**0.001**	− 0.01 (− 0.02 to 0.00)	0.110
Former smoking vs never smoking	0.17 (− 0.02 to 0.37)	0.075	0.02 (− 0.28 to 0.33)	0.876
Current smoking vs never smoking	0.35 (0.06 to 0.65)	**0.020**	0.23 (− 0.16 to 0.62)	0.255
HDL cholesterol	0.21 (− 0.01 to 0.44)	0.059	− 0.16 (− 0.54 to 0.23)	0.428
Total cholesterol	− 0.21 (− 0.31 to − 0.11)	** < 0.001**	− 0.06 (− 0.21 to 0.08)	0.384
Diabetes mellitus	− 0.08 (− 0.40 to 0.24)	0.626	− 0.01 (− 0.40 to 0.39)	0.980
BP lowering medication	− 0.05 (− 0.25 to 0.16)	0.658	− 0.27 (− 0.55 to 0.02)	0.064
Lipid-reducing medication	− 0.43 (− 0.67 to − 0.19)	** < 0.001**	− 0.11 (− 0.45 to 0.23)	0.520

*BMI* body mass index, *WC* waist circumference, *BP* blood pressure, *DBP* diastolic blood pressure, *SBP* systolic blood pressure, *HDL* high-density lipoprotein. *P* values in bold indicate statistical significance at *p* < 0.05 level.

*The knot was median age, 67.0 years for women and 66.6 years for men by default.

**In women, effect estimate (95% CI) of SBP was − 0.011 (− 0.018 to − 0.004) and − 0.008 (− 0.018 to 0.002) in men.

## Discussion

Using data from the large population-based Rotterdam Study, we provide reference distributions of absolute and BSA-adjusted IAD by age and sex categories. We further report on determinants of IAD among elderly women and men.

Aortic size is influenced by age, sex, race, body habitus and aortic measurement locations. Studies providing normal IAD reference values are sparse, and no prior study has reported ultrasound-based reference values for IAD in European population. The Framingham Heart Study and the Copenhagen General Population Study reported normal values of IAD based on CT measurement among the general population in their fifties^3,4^. Another MRI-based study was performed among participants aged 20–79 years^10^. Compared with these studies, our reported reference values are slightly smaller at the corresponding age categories. Part of the differences may be caused by the use of different measurement methods, although no consensus regarding the comparison of aortic diameters based on different measurement modalities exist^17,18^. Joh et al.^19^ reported ultrasound-based reference values for abdominal aortic diameter among an elderly Korean population. In their study, mean IAD was 17.9 mm in women, which is 0.9 mm larger than our study, and 19.0 mm in men, which is 0.5 mm smaller than our study.

We found upper percentiles (75th, 90th, 95th) of IAD to increase with age, while the changes of lower percentiles (5th, 25th) were negligible. This phenomenon indicates that aortic dilatation is uneven in the general population^20^. This hypothesis is also supported by previous studies showing that people with larger baseline diameters were prone to aortic dilatation and possibly developing an AAA^21–23^. It is noticeable that the BSA-adjusted diameter percentiles increase more evenly with advancing age. This suggests that the individual variation is reduced in anthropometric-corrected diameters. Nevertheless, our study has a cross-sectional nature, and therefore more prospective studies are needed to explore trajectories of IAD growth over time.

The non-linear relation between age and IAD is an interesting finding in our study. We showed that the diameter increases sharply up to 70 years of age after which the increase slows down, and the IAD reaches a plateau around 75 years in both sexes. Biologically, age-related aortic expansion is thought to be related to aortic wall remodeling regarding fragmentation of the elastin fibers, increased synthesis of collagen, aortic stiffness, and calcification^24,25^. However, the underlying mechanism needs further investigation. Of note, our study excluded participants with IAD ≥ 30 mm. Individuals over 70 years of age are more likely to have an IAD larger than 30 mm.

In addition to age, this study further confirmed that obesity is a risk factor for aortic expansion^2,4,20,26^. Although BMI and WC are both obesity-related anthropometric indicators, WC provides more information on abdominal obesity, while BMI represents systemic obesity. In our study, larger WC was associated with larger IAD in both sexes, while BMI was associated with IAD mainly in women. Our results therefore suggest that increased visceral adiposity in both sexes and increased BMI in women calls for careful consideration in AAA screening programs as these individuals might be more prone to infrarenal aortic dilatation.

Previous studies suggested DBP to be a stronger risk factor for aortic dilatation than SBP^23,27^. Our study observed that higher diastolic, but lower systolic, blood pressure were associated with larger IAD. Arterial stiffness that protects against AAA might account for the stronger association with DBP and weaker association with SBP^27,28^. Mensel et al.^10^ also observed a positive association of DBP with larger BSA-adjusted IAD but an inverse association with SBP. Our study found DBP to be a risk factor for BSA-adjusted IAD mainly in women while SBP had no significant association with adjusted IAD in both sexes.

Smoking is well proved to be a risk factor for larger abdominal aortic diameter^10,13,26,29,30^. However, we did not find a significant association between smoking behavior and enlarged IAD in men. This may have been due to the fact that more than half of the men in our population (57.9%) were former smokers and we did not have detailed information on their smoking cessation. Besides, information on pack-years of smoking was not available. Of note, smoking carried a greater risk for IAD expansion among women than men in our study, which is consistent with previous studies^12,29^. We speculate that absorption or pathophysiological impact of toxins from cigarette smoking might be greater in women. Similarly, female smokers are more likely to develop lung cancer^31^ and heart disease^32^ compared to male smokers. Overall, it is an encouraging finding to advocate smoking cessation, especially in women, for preventing abdominal aortic dilatation.

Total cholesterol was also inversely associated with infrarenal aortic dilatation among women in this study, which is in contrast to previous studies^10,33^. HDL showed to be positively significantly related to BSA-adjusted IAD in this study. The association of favorable lipid profile with larger aortic diameter is a paradoxical finding. Although we took the impact of lipid lowering medication into account, such inverse association could still be, at least partly, due to other factors which might be potential confounders, such as genetic factors^34^.

This is the first study reporting age- and sex-specific ultrasound-based reference values for IAD among the non-aneurysmal European general population. Strengths of the current study include a large sample size, per protocol assessment of IAD by the same technician in the whole study, and availability of meticulously measured various cardiovascular risk factors. Our study provides sex-specific information regarding reference values and risk factors of enlarged diameters among a well-defined non-aneurysmal population. The potential limitations of this study also merit attention. First, although all measurements were performed by professional technicians under a standard protocol, ultrasound measurement remains to be highly operator dependent, and inter- and intra-observer variability exists. Second, given the inherent limitations of a cross-sectional study, no inferences regarding causality can be made. Third, our study population was merely of European ancestry and therefore our results might not be generalizable to other ethnicities.

In conclusion, we presented ultrasound-based age- and sex-specific reference values of IAD among the middle-aged and elderly European population. The differences in IAD references value between women and men diminished after taking BSA into account. Age showed a non-linear association with larger abdominal aortic diameter. The increase in diameters was attenuated after 70 years. We also showed sex differences in the associations of traditional cardiovascular risk factors with IAD, albeit the sex differences were not statistically significant.

## Supplementary Information

Supplementary Information.

## Data Availability

Rotterdam Study data can be made available to interested researchers upon request. Requests can be directed to data manager Frank J.A. van Rooij (f.vanrooij@erasmusmc.nl) or visit the following website for more information http://www.ergo-onderzoek.nl/wp/contact. We are unable to place data in a public repository due to legal and ethical restraints. Sharing of individual participant data was not included in the informed consent of the study, and there is potential risk of revealing participants’ identities as it is not possible to completely anonymize the data.
