# Supplementary material for: Sex-specific normal values and determinants of infrarenal abdominal aortic diameter among non-aneurysmal elderly population
Source: Sci Rep. 2021 Sep 7;11:17762. doi: 10.1038/s41598-021-97209-3 (PMC8423780; doi:10.1038/s41598-021-97209-3)
Supplement: Supplementary file 1 — Supplementary Information. [file 41598_2021_97209_MOESM1_ESM.pdf]

Supplementary material

Title: Sex-specific normal values and determinants of infrarenal abdominal aortic diameter among non-aneurysmal elderly population

Fang Zhu [1], Banafsheh Arshi [1], M. Arfan Ikram [1], Robert J. De Knegt [1, 2], Maryam Kavousi [1]

[1] Department of Epidemiology, Erasmus MC, University Medical Center Rotterdam, Rotterdam, the Netherlands

[2] Department of Gastroenterology & Hepatology, Erasmus MC, University Medical Center Rotterdam, Rotterdam, the Netherlands

Supplementary Table S1. Univariate associations between cardiovascular risk factors and absolute infrarenal abdominal aortic diameters among women and men

	Women		Men	
Risk factors	Effect estimate (95% CI)	*p*	Effect estimate (95% CI)	*p*
Age	0.07 (0.06 to 0.08)	<0.001	0.08 (0.06 to 0.09)	<0.001
BMI	0.1 (0.07 to 0.12)	<0.001	0.09 (0.05 to 0.13)	<0.001
WC	0.04 (0.03 to 0.05)	<0.001	0.04 (0.02 to 0.05)	<0.001
DBP	0.03 (0.03 to 0.04)	<0.001	0.02 (0.01 to 0.03)	0.004
SBP	0.02 (0.01 to 0.02)	<0.001	0.02 (0.01 to 0.02)	<0.001
Former smoking[+]	0.12 (-0.08 to 0.33)	0.234	0.39 (0.07 to 0.7)	0.016
Current smoking[+]	-0.09 (-0.39 to 0.22)	0.577	0.21 (-0.2 to 0.61)	0.311
HDL cholesterol	-0.21 (-0.42 to 0.01)	0.06	-0.2 (-0.57 to 0.16)	0.279
Total cholesterol	-0.16 (-0.26 to -0.07)	0.001	-0.1 (-0.23 to 0.02)	0.108
Diabetes mellitus	0.34 (0.02 to 0.66)	0.036	0.36 (-0.02 to 0.74)	0.066
Blood pressure lowering medication	0.36 (0.16, 0.56)	<0.001	0.18 (-0.09, 0.45)	0.187
Lipid-reducing medication	-0.01 (-0.34 to -0.13)	0.374	0.08 (-0.23 to 0.39)	0.596

[+]Compared with never smokers.

BMI, body mass index; WC, waist circumference; DBP, diastolic blood pressure; SBP, systolic blood pressure; HDL, high-density lipoprotein.

Supplementary Table S2. Associations between cardiovascular risk factors and body surface area-adjusted infrarenal aortic diameters among women and men

	**women**		**men**	
Risk factors	Effect estimate (95% CI)	*p*	Effect estimate (95% CI)	*p*
(Intercept)	8.12 (7.54 to 8.71)	**<0.001**	8.23 (7.53 to 8.92)	**<0.001**
Age [1st]*	3.47 (3.05 to 3.89)	**<0.001**	3.68 (3.16 to 4.20)	**<0.001**
Age [2nd]*	2.20 (1.70 to 2.70)	**<0.001**	2.02 (1.41 to 2.63)	**<0.001**
DBP	0.01 (0.00 to 0.01)	0.051	0.00 (-0.01 to 0.01)	0.488
SBP	0.00 (-0.01 to 0.0)	0.138	0.00 (-0.01 to 0.00)	0.493
former smoke†	0.05 (-0.07 to 0.17)	0.414	-0.02 (-0.19 to 0.14)	0.765
current smoke†	0.34 (0.16 to 0.52)	**<0.001**	0.23 (0.02 to 0.43)	**0.029**
HDL cholesterol	0.40 (0.27 to 0.53)	**<0.001**	0.32 (0.13 to 0.51)	**0.001**
Total cholesterol	-0.10 (-0.16 to -0.04)	**<0.001**	-0.06 (-0.13 to 0.02)	0.134
Diabetes mellitus	-0.15 (-0.34 to 0.05)	0.134	-0.13 (-0.34 to 0.08)	0.218
Blood pressure lowering medication	-0.20 (-0.32 to -0.08)	**0.001**	-0.21 (-0.36 to -0.06)	**0.001**
Lipid-reducing medication	-0.19 (-0.33 to -0.04)	**0.012**	-0.06 (-0.24 to 0.12)	0.511

* The cut knot was median age, 67.0 for women and 66.6 for men by default.

†Compared with never smoke.

SBP, systolic blood pressure; DBP, diastolic blood pressure; HDL, high-density lipoprotein.

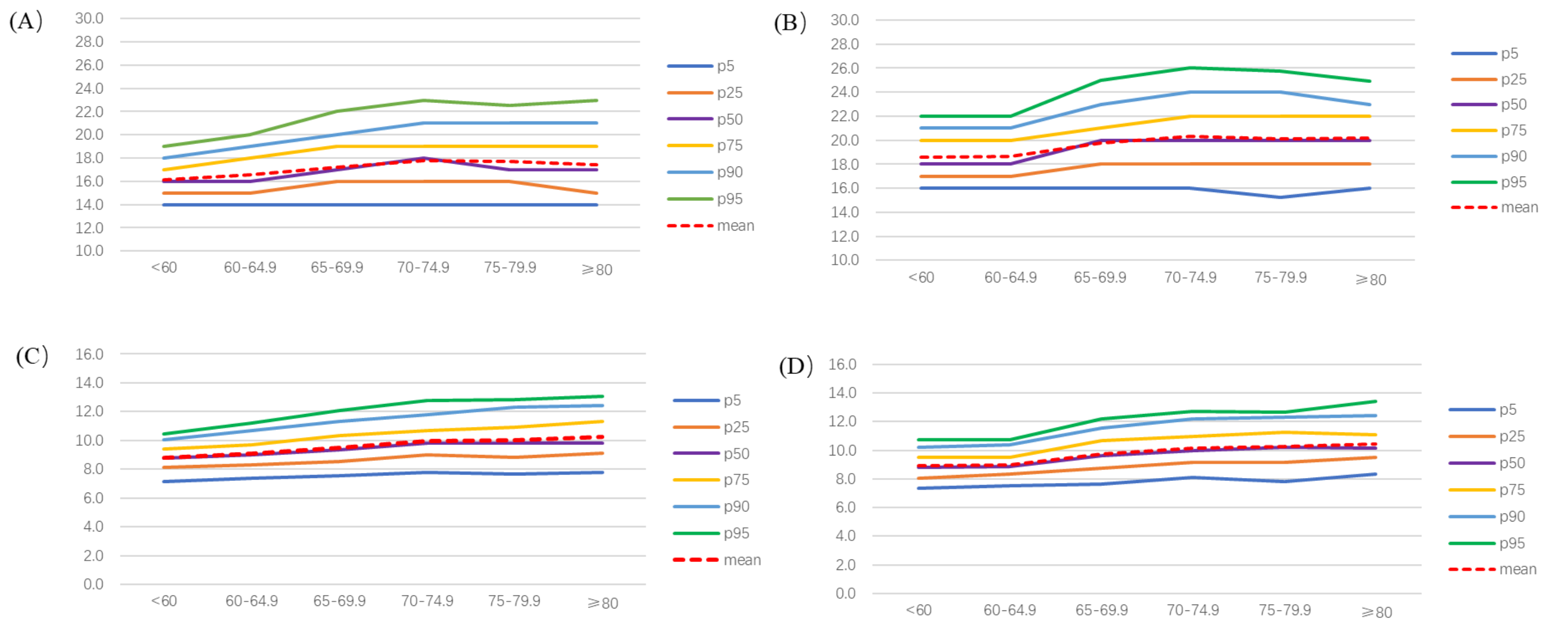

Supplementary Figure S1(A) Absolute infrarenal aortic diameter value changes among different age groups (women); (B) Absolute infrarenal aortic diameter value changes among different age groups (men); (C) Body surface area-adjusted diameter value changes among different age groups (women); (D) Body surface area-adjusted diameter value changes among different age groups (men);
